# Overshoot mechanism in transient excitation of THz and Gunn oscillations in wide-bandgap semiconductors

**DOI:** 10.1186/1556-276X-7-647

**Published:** 2012-11-24

**Authors:** Ernesto Momox, Nick Zakhleniuk, Naci Balkan

**Affiliations:** 1 School of Computer Science and Electronic Engineering, University of Essex, Wivenhoe Park, Colchester CO4 3SQ, UK

**Keywords:** Gunn diode, THz, Wide-bandgap semiconductors, Drift-diffusion, Hot electrons, Electron temperature, Device simulation, 07.05.Tp, 85.30.Fg, 77.84.Bw

## Abstract

A detailed study of high-field transient and direct-current (DC) transport in GaN-based Gunn diode oscillators is carried out using the commercial simulator Sentaurus Device. Applicability of drift-diffusion (DD) and hydrodynamic (HD) models to high-speed, high-frequency devices is discussed in depth, and the results of the simulations from these models are compared. It is shown, for a highly homogeneous device based on a short (2 μm) supercritically doped (10^17^ cm^−3^) GaN specimen, that the DD model is unable to correctly take into account some essential physical effects which determine the operation mode of the device. At the same time, the HD model is ideally suited to solve such problems due to its ability to incorporate non-local effects. We show that the velocity overshoot near the device contacts and space charge injection and extraction play a crucial role in defining the operation mode of highly homogeneous short diodes in both the transient regime and the voltage-controlled oscillation regime. The transient conduction current responses are fundamentally different in the DD and HD models. The DD current simply repeats the velocity-field (v-F) characteristics, and the sample remains in a completely homogeneous state. In the HD model, the transient current pulse with a full width at half maximum of approximately 0.2 ps is increased about twofold due to the carrier injection (extraction) into (from) the active region and the velocity overshoot. The electron gas is characterized by highly inhomogeneous distributions of the carrier density, the electric field and the electron temperature. The simulation of the DC steady states of the diodes also shows very different results for the two models. The HD model shows the trapped stable anodic domain in the device, while the DD model completely retains all features of the v-F characteristics in a homogeneous gas. Simulation of the voltage-controlled oscillator shows that it operates in the accumulation layer mode generating microwave signals at 0.3 to 0.7 THz. In spite of the fact that the known criterion of a Gunn domain mode *n*_0_*L* > (*n*_0_*L*)_0_ was satisfied, no Gunn domains were observed. The explanation of this phenomenon is given.

## Background

At present, there is a considerable interest in development of compact emitters of high-frequency radiation in the terahertz (THz) and sub-THz spectral range. Various physical mechanisms of THz generation have been suggested and investigated in recent years. Most of these mechanisms rely on either purely optical THz radiation generation (e.g. CO_2_ laser-pumped THz molecular lasers) or combination of optical and electrical excitation (e.g. using laser difference frequency photomixing or laser-excited uni-travelling carrier photodetectors). Development of compact sources of THz radiation with purely electronic excitation still presents a formidable challenge. This is possibly in part due to the peculiar location of the THz spectrum which is between the optical and microwave (MW) regions. The aforementioned spectral regions can easily be accessed using optical sources (lasers) or electronic microwave emitters, respectively.

One of the promising physical mechanisms in semiconductors, which is capable in principle of generating the MW and possibly THz radiation, is a Gunn effect due to negative differential mobility (NDM) caused by the transferred electron effect in strong electric fields [1]. Until very recently, the research and development of Gunn MW oscillators was focused on utilization of traditional III-V semiconductors, like GaAs, and their compounds. The maximum emission frequency in these III-V emitters is limited to a few 100 GHz at best.

Recent successes in technology of III-V nitrides (in particular, GaN, InN, AlN and their compounds) led to development of a range of novel optoelectronic and microelectronic devices based on these materials which show superior characteristics. Wide-bandgap nitrogen-based semiconductors are also becoming attractive candidates for electronic generation of THz radiation. Due to non-monotonous velocity-field (v-F) characteristics, record-high peak velocities and very high breakdown fields, these materials are capable of generating high-power, high-frequency radiation, as was reported recently from numerical simulations [2,3] and the first experimental observation [4]. Depending on the operation mode, Gunn diodes usually work at a fixed direct-current (DC) bias or a combination of DC and alternating-current (AC) biases [1]. In the past few years, the attention also turned to transient regimes where a short (≤1 ps) high-electric field pulse is incident on a sample with the NDM, using, e.g. a photoconductive switch as in [5]. In the latter case, it is assumed that the electron gas remains spatially homogeneous and there is no space charge injection, contrary to what usually takes place in a DC (or DC + AC)-driven Gunn diode.

In this paper, we first investigate numerically transient and DC regimes of the electron transport in GaN samples and then study operation of GaN-based Gunn diode oscillators and their potential for generating MW and THz radiation using Sentaurus Device software from Synopsys (Mountain View, CA, USA) [6]. The first part of this study is motivated by the necessity to evaluate and compare various transport models and to justify their choice and use in simulating the Gunn diodes in DC and transient regimes. It also gives a good insight into the underlying physics of the device operation. In the second part of the paper, the operation of the DC-driven GaN-based Gunn oscillators is investigated, and it is shown that the short supercritically doped diodes operate in the accumulation layer mode at very high (near THz) frequencies. The obtained results are summarised in the final section.

## Methods

### DC and transient transport and simulation models

Typical regimes of Gunn diode operation are characterized by highly inhomogeneous spatial-temporal distributions of non-equilibrium carriers and fields. Charge excitations (domains or accumulation layers) propagate from the cathode through the active region of the specimen and generate current or voltage pulses when they are discharged at the anode, or in some cases, they may be transformed into the stationary anodic domains [1,7,8]. It is well known that in many cases, the drift-diffusion (DD) model gives a very good and correct semi-quantitative description of various Gunn diode regimes [9]. The key physical feature of DD models is the instantaneous dependence of all kinetic coefficients which describe carrier transport (mobility, diffusion coefficient, etc.) on the local electric field or the gradient of quasi-Fermi potential. The hot-electron effects are principally absent from the DD models. The carrier mean energy is solely determined by the lattice temperature and does not depend on the electric field, although the kinetic coefficients are in general taken as field-dependent.

At the same time, as was first pointed out in [10], if there is a sufficiently rapid variation of the electric field with distance and/or time, then the field dependence of the kinetic coefficients may be non-local and non-instantaneous. This means that the relaxation effects must be taken into account. This point is particularly of great importance in the devices operating at high frequencies (usually, higher than a few 10 GHz). All relevant relaxation processes are by definition included in the microscopic description of the carrier transport based on the Boltzmann equation. However, since the Boltzmann equation is notoriously difficult to solve in the case of spatially and time-dependent fields, this approach is not suitable for direct applications to most semiconductor devices. Instead, one has to use the simplified set of macroscopic continuity equations which follow from the Boltzmann equation by averaging (integration). The simplest set of the equations is the one which consists of a single carrier density continuity equation (together with the expression for the conduction current). This is the already mentioned DD model. The next set is the one consisting of both equations, which are the carrier density continuity equation and the mean carrier energy continuity (balance) equation (together with the equations for the current density and the energy flux density). In the case when the carrier mean energy can be described by the electron temperature, the model in question is called the hydrodynamic (HD) model. Although the HD model is more difficult to implement and it does not provide a comprehensive description of all possible non-equilibrium effects, it does have at least two new physical features which are very important for device modelling. Firstly, it describes the non-local field dependence of the kinetic coefficients which now depend on the electron temperature rather than on the electric field as in the case of the DD model. Due to the carrier and energy flows from one region of a device to the other, the local electron temperature at a given point depends on inhomogeneous distributions of the carrier density and the electric field over the whole device. Secondly, the HD model describes the spatial-temporal hot-electron energy transport, which in turn takes into account the carrier energy relaxation effects, i.e. the kinetic coefficients are not anymore the instantaneous functions of the electric field. If the electric field varies with time, then due to the finite value of the carrier energy relaxation time, the electron temperature at a given moment of time will also depend on the values of the electric field taken in the previous moments of time. In addition, the thermal diffusion contribution to the carrier transport is now described self-consistently. As a result of the inclusion of the above two features into the HD model, the model is able to tackle the spatial and temporal overshoot effects which play a very important role in high-speed semiconductor devices [9].

## Results and discussion

### Transient response of GaN samples to ultra-short high-electric field pulses

In order to investigate the differences in applying the DD and HD models to simulation of the high-field transport in high-speed devices, we will first study the transient response of a GaN sample induced by high-electric field ultra-short pulses. We assume that the GaN sample is connected in series to a photoconductive switch and the external DC bias is applied to these two components. Due to the extremely high resistivity of the open-state switch, practically all applied bias will drop on the switch. When the switch is optically activated (switched to a closed state) by an ultra-short laser pulse, its resistivity drops below the GaN sample resistivity, and practically all external bias will be applied to the sample for the duration of the closed state of the switch. In this way, it is possible to actually apply a very short bias pulse to the contacts of the sample, which in turn will generate the high-electric field pulse in it. Similar setups are used, for example, in photoconductive switching of DC-biased strip lines [10-12] where the desired output signal is the current pulse which is directly generated by the switch. Here, we use the switch as the gate generating the short voltage pulse applied to the contacts of a GaN sample and wish to calculate the current response of the sample. Our case is also different from the one which was investigated in [5] where the very short MW transient field generated by a photoconductive switch was incident on a thin short-circuited GaN plate, entirely illuminating it. The principal difference from our case is that in [5] there was no external bias applied to the sample contacts, i.e. there was no carrier injection from the contacts and no space charge or diffusion effects present, and the drift current simply follows the v-F characteristics at a constant carrier density which is defined by the doping.

### The DD model

Sentaurus Device is a 2D-3D simulator which is able to model various devices with complex geometry. However, in our case of a Gunn diode, it is sufficient to consider a 1D geometry. We consider a GaN specimen of equal length *L* and width *W*, *L = W =* 2 μm. The cathode is at *x* = 0, and the anode at *x* = *L*. The software takes the default thickness of *d =* 1 μm along the third direction, so the cross section-dependent parameters (e.g. the current) can be easily scaled with *d*.

In the DD modelling, the device is described by the continuity equation for the carrier density *n*(*x*,*t*) and the electron current density *j*_*n*_(*x*,*t*):

(1) $$\frac{\partial n\left(x,t\right)}{\partial t}+\frac{1}{e}\frac{\partial j_{n}\left(x,t\right)}{\partial x}=0\text{,}$$

the Poisson equation for electric field *E*(*x*,*t*):

(2) $$\frac{\partial E\left(x,t\right)}{\partial x}=\frac{e}{\varepsilon \varepsilon_{0}}\left[n_{0}\left(x\right)-n\left(x,t\right)\right]\text{,}$$

and the expression for current density *j*_*n*_(*x*,*t*):

(3) $$\begin{array}{rl} j_{n}\left(x,t\right)= & en\left(x,t\right)\mu_{n}\left(E\right)E\left(x,t\right)\\ & +eD_{n}\left(E\right)\left[\frac{\partial n\left(x,t\right)}{\partial x}-n\left(x,t\right)\frac{\partial \ln\gamma_{n}\left(x,t\right)}{\partial x}\right]\text{.} \end{array}$$

Here, *t* is the time, *e* is the electron charge, *ε* is the dielectric constant of GaN (*ε* = 9.5), *ε*_0_ is the permittivity of free space, *n*_0_(*x*) = *N*_D_(*x*) is the doping density, *μ*_*n*_(*E*) is the electron mobility dependent on the local instantaneous electric field and *D*_*n*_(*E*) is the field-dependent diffusion coefficient.

Although the software allows us to compute *D*_*n*_(*E*) independently using a physical model interface (PMI), we will use the default option in which the diffusion coefficient is given through the mobility by the Einstein relation, *D*_*n*_(*E*) = (*k*_0_*T*/*e*)*µ*_*n*_(*E*), where *k*_0_ is the Boltzmann constant and *T* is the ambient temperature which will be assumed here as *T* = 300 K. It is well known that the Einstein relation is not satisfied at non-equilibrium conditions and definitely gives inaccurate results at intermediate fields (see, e.g. the results of [13] for GaAs and InP), but the Einstein relation still gives qualitatively correct dependence of *D*_*n*_(*E*) at high electric fields which are of the main interest for us. Another problem with *D*_*n*_(*E*) is that at present there are little reliable data for non-equilibrium diffusivity in GaN.

The last term in the square brackets in Equation 3 takes into account the effect of the electron gas degeneracy (Fermi statistics) on the diffusion current with *γ*_*n*_(*x*, *t*) = [*n*(*x*, *t*)/*N*_*c*_]exp[−*η*_*n*_(*x*, *t*)], where *N*_*c*_ is the electron effective density of states, *η*_*n*_(*x*, *t*) = [*E*_F_(*x*, *t*) − *E*_*c*_(*x*, *t*)]/*k*_0_*T*], *E*_F_(*x*,*t*) is the local electron quasi-Fermi level and *E*_*c*_(*x*,*t*) is the local conduction band edge. For Boltzmann statistics, *γ*_*n*_ = 1.

Regarding the non-equilibrium carrier mobilities, the software has two main options for the DD transport:

(a) The transferred electron (TE) model [6]:

(4) $$\mu_{n}\left(E\right)=\frac{\mu_{0}+\frac{v_{s}}{E}{\left(\frac{E}{E_{0}}\right)}^{4}}{1+{\left(\frac{E}{E_{0}}\right)}^{4}}\text{,}$$

(b) The Canali (CA) model [6]:

(5) $$\mu_{n}\left(E\right)=\frac{\mu_{0}}{{\left[1+{\left(\frac{\mu_{0}E}{v_{s}}\right)}^{\beta }\right]}^{1/\beta }}\text{.}$$

Here, *μ*_0_ is the low-field mobility, *v*_s_ is the saturation drift velocity, *E*_0_ is the threshold field and *β* is a fitting parameter. As of now, there is a considerable uncertainty in the literature regarding experimental and theoretical results for non-equilibrium v-F characteristics in GaN (see, e.g. discussion in [14] and the references therein). Here, we will use the following parameters: *μ*_0_ = 700 cm^2^/(V· s), *v*_s_ = 2 × 10^7^ cm/s, *E*_0_ = 100 kV/cm and *β* = 2. These material parameters are very close to the parameters used for the zinc-blende GaN Gunn diode in [15]. Regarding the active region of the diode, we assume that the sample is homogeneously doped with *n*_0_ = 10^17^ cm^−3^ and that the cathode and the anode are metallic contacts, i.e. we do not use the heavily doped *n*^+^ contact regions near the electrodes [9]. There is a subtle issue regarding the effect of the type of contacts on the Gunn diode operation regimes which warrants a separate investigation. This will be presented elsewhere and will not be investigated here. Also, we do not introduce the doping notch near the cathode, which is usually required to nucleate the Gunn domains [1], for the same reasons.

**Figure 1:**
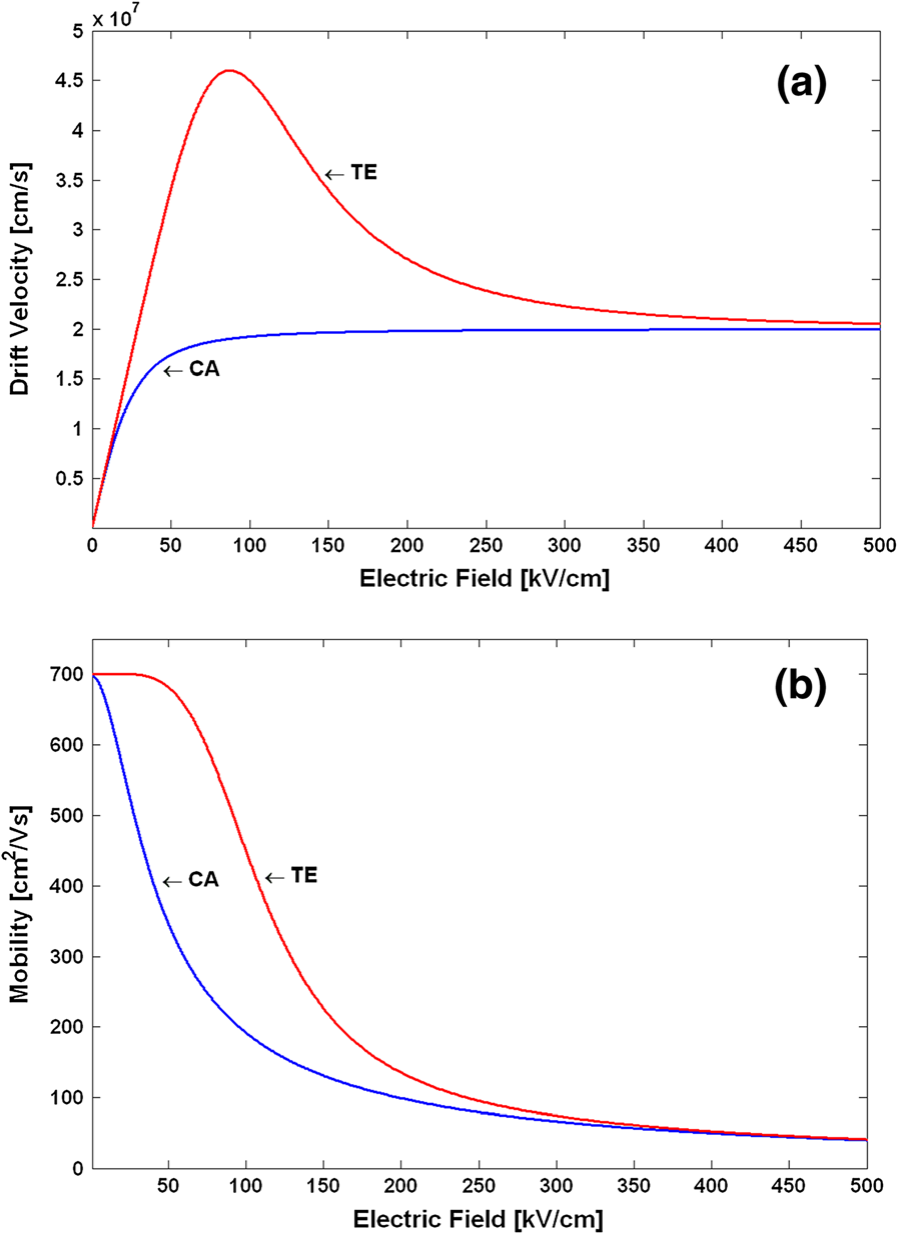
**Velocity-field characteristics (a) and field-dependent mobilities (b) of GaN samples for various models.** TE - transferred electron model, CA - Canali model.

**Figure 2:**
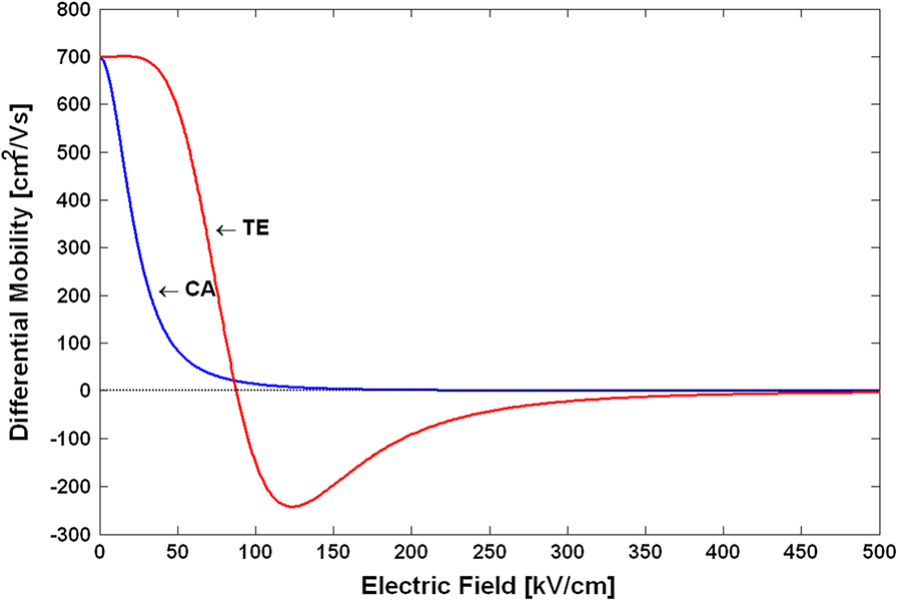
Differential mobilities of GaN as functions of the electric fields.

The v-F characteristics and the *μ*_*n*_(*E*) dependences for the TE and CA models in GaN are shown in Figure 1a,b, respectively, using the above parameters and Equations 4 and 5. The differential mobilities *µ*_d_ = *dv*_d_/*dE* were also calculated and are shown in Figure 2. These dependences are used in the subsequent analysis of the GaN Gunn diode operation. In particular, we need to know the absolute values of the NDM (|*μ*_d_(*E*)|) to use it in the criterion which separates the various modes of operation [1,9]. As can be seen from Figure 2, the peak of |*μ*_d_(*E*)| in GaN is about 250 V/(cm^2^· s). This value is about an order of magnitude smaller than the |*μ*_d_(*E*)| in GaAs. Our results also agree well with the NDM calculated in [15] for a zinc-blende GaN. Using the obtained non-equilibrium local mobilities *μ*_*n*_(*E*) in the basic DD model equations (Equations 1 to 3), one can now simulate the spatial-temporal distributions of the carrier density *n*(*xt*) and the electric field *E*(*xt*). This is the end result of the DD model, which allows us to calculate all the parameters and characteristics of the device operation, like transient responses or the current–voltage (*IV*) characteristics for a stationary operation.

### The HD model

In the HD model, the energy balance equation is added to the above Equations 1 and 2. The HD model assumes that the carrier temperature concept can be applied to a non-equilibrium electron gas. This in turn requires that the electron–electron (e-e) scattering rate in the material is faster than the electron energy relaxation rates. This condition can be satisfied at high carrier densities (typically, at least above 10^16^ cm^−3^). The high carrier densities can be provided either by doping or by the carrier injection from the contacts. As far as the Gunn diodes are concerned, this point requires some further physical discussion.

First of all, there are at least two groups of electrons in materials with III-V band structure [9], the upper valley heavy-effective-mass electrons and the lower valley light-effective-mass electrons, with dynamic inter-valley transfer of the electrons. Since population of these groups depends on the applied electric field and the electrons in different groups respond differently to this field (they have different degrees of deviation from the equilibrium in terms of their mean energy), in principle, one has to consider each group separately with the corresponding carrier density and the electron temperature.

There were a number of attempts to develop a many-valley HD model of Gunn oscillators (see, e.g. [16]) since the first one-temperature HD theory [17]. The key problem here is that it is very difficult to provide *a priori* known and reliable input kinetic coefficients for macroscopic equations for each carrier group at non-equilibrium conditions. The best outcome achieved with many-valley HD models was the better insight into the relaxation processes and the clear and important proof [18] that if the electron temperature distributions in the device are spatially inhomogeneous, then the HD models are capable at least at a qualitative or a semi-quantitative level to correctly tackle contributions from spatial-temporal relaxation processes which are outside of the scope of the DD models. The latter is the most important and novel element of HD models which allows to justifiably apply them to Gunn diodes working at very high frequencies (of the order of the carrier energy relaxation rates).

The HD models are also helpful in calculating the static v-F characteristics [1] in homogeneous non-equilibrium gas. It is well known [19,20] that the static v-F characteristics which were calculated within the HD approach remain valid even in the situations when the HD model is physically inapplicable, e.g. at very low electron densities. Although this peculiar property of the HD model has not been explained or proved so far, most likely it has something to do with the shape of non-equilibrium distribution functions used (the Maxwell-Boltzmann or Fermi functions, which differ from the equilibrium distributions by substitution of the lattice temperature by the electron temperature). Note that it is more difficult to justify the use of the displaced Maxwellian or Fermi functions [19] which are described by two parameters - the electron temperature and the drift wave vector, as this requires a complete e-e control of the energy and momentum relaxation. This is practically impossible to achieve in bulk semiconductors since the required high doping in order to obtain high carrier density also results in high electron momentum scattering rates by the doping impurities, i.e. the e-e and momentum scattering rates are of the same order. Note also that the use of displaced distribution functions means that one more equation (which is the average momentum balance equation) should be added in the HD model. This in turn requires more kinetic coefficients to be known *a priori* and to be used as the input parameters in the extended model. From this point of view, the HD models based on the electron temperature are better physically justified and more computationally effective for the device modelling. The additional physical support and justification for the HD models in the case of the GaN-based devices investigated here is that the level of doping is around 10^17^ cm^−3^. The corresponding carrier densities are high enough to warrant the introduction and the use of the electron temperature.

Currently all available commercial simulation tools, to the best of our knowledge, use the HD models with a single temperature assigned to the same type of carriers (e.g. the electrons and the holes, as different types of carriers, may have different temperatures). The above discussion and the analysis of vast literature on device simulations show that these models provide good insight into the underlying physics, correctly describe various working regimes and give a good agreement between the simulation results and the available experimental data.

In the HD model, Equations 1 and 2 are supplemented by the energy balance equation [6]:

(6) $$\begin{array}{rl} \frac{\partial W_{n}\left(x,t\right)}{\partial t}+\frac{\partial S_{n}\left(x,t\right)}{\partial x}= & ej_{n}\left(x,t\right)E\left(x,t\right)\\ & -\xi_{n}\frac{W_{n}\left(x,t\right)-W_{n0}}{\tau_{\varepsilon }}\text{,} \end{array}$$

where the energy flux density

(7) $$S_{n}\left(x,t\right)=-\frac{5r_{n}\lambda_{n}}{2}\left\lceil \frac{k_{0}T_{n}\left(x,t\right)}{e}j_{n}\left(x,t\right)+f_{n}^{\mathrm{hf}}K_{n}\frac{\partial T_{n}\left(x,t\right)}{\partial x}\right\rceil \text{,}$$

where *κ*_*n*_ = (*k*_0_^2^/*e*)*n*(*x*, *t*)*µ*_*n*_*T*_*n*_(*x*, *t*) is the heat conductivity, *λ*_*n*_ is expressed via the Fermi integrals *λ*_*n*_ = *F*_1/2_(*η*_*n*_)/*F*_− 1/2_(*η*_*n*_) (*λ*_*n*_ = 1 for Boltzmann statistics), *T*_*n*_(*x*, *t*) is the local electron temperature, *W*_*n*_(*x*, *t*) = (3/2)*n*(*x*, *t*)*k*_0_*T*_*n*_(*x*, *t*) and *W*_*n*0_(*x*, *t*) = (3/2)*n*(*x*, *t*)*k*_0_*T* are the local carrier energy densities at different temperatures, and *τ*_*ε*_ is the energy relaxation time. Sentaurus Device has a PMI which allows in general to specify arbitrary temperature dependence of *τ*_*ε*_. Since this frequently introduces serious convergence problems and because at present no reliable energy-dependent relaxation time is available, we will use in our simulations a constant value *τ*_*ε*_ = 0.1 ps for GaN. It is well known from the experiments [21,22] and from various theoretical and numerical calculations [23] that the energy relaxation rate in GaN is at least an order of magnitude higher than in GaAs, and it has a tendency to further increase when the electron energy increases. The parameter *ξ*_*n*_ in Equation 6 improves numerical stability [6].

The electron current density in the HD model differs from Equation 3 for the DD model:

(8) $$\begin{array}{rl} j_{n}\left(x,t\right)= & e\mu_{n}\left(T_{n}\right)[n\left(x,t\right)E\left(x,t\right)\\ & +f_{n}^{\mathrm{td}}\lambda_{n}k_{0}n\left(x,t\right)\frac{\partial T_{n}\left(x,t\right)}{\partial x}\\ & +\frac{k_{0}T_{n}\left(x,t\right)}{e}\left(\frac{\partial n\left(x,t\right)}{\partial x}-n\left(x,t\right)\frac{\partial \ln\gamma_{n}\left(x,t\right)}{\partial x}\right)] \end{array}$$

Note that the Einstein relation was used in deriving Equation 8, although Sentaurus Device provides the PMI to compute the diffusion coefficients independently [6], as was already mentioned. The second term in Equation 8 takes into account the thermal diffusion contribution to the current density.

The three numerical parameters in Equations 7 and 8, *r*_*n*_, *f*_*n*_^*td*^ and *f*_*n*_^*hf*^, allow an independent variation of the convective and the diffusive contributions in the energy flux density and the current density. The preferable choices for these parameters are *r*_*n*_ = 3/5, *f*_*n*_^*td*^ = 0 and *f*_*n*_^*hf*^ = 1 (this corresponds to the so-called Stratton model [24], the default option) and *r*_*n*_ = 1, *f*_*n*_^*td*^ = 1 and *f*_*n*_^*hf*^ = 1 (this corresponds to the so-called Blotekjaer model [25]). In the present work, we use the Stratton model, although our investigation shows that the choice of the values of the above parameters does affect the output results for some regimes of the Gunn diode. Since there is no universal rule regarding the choice of the fitting parameters, an adequate choice can only be made on the basis of deep physical analysis of the model and the processes in the studied device and by comparing the simulation results against experimental data.

The electron temperature-dependent mobilities *μ*_*n*_(*T*_*n*_) in the HD model in Sentaurus Device are obtained from Equations 4 and 5 using the following approach. It is assumed that the v-F characteristics obtained for a homogeneous non-equilibrium steady state remain valid in a non-equilibrium system with inhomogeneous and instantaneous fields. In this case, the local energy balance equation in a single-electron approximation *eµ*_*n*_(*T*_*n*_)*E*^2^ = (3/2)*k*_0_[*T*_*n*_ − *T*]/*τ*_*ε*_ can be solved for the electric field *E*. The obtained field is substituted into Equations 4 and 5 which are then solved for *μ*_*n*_ as a function of *T*_*n*_.

In the case of the CA model, Equation 5 can be solved analytically [6] to give

(9) $$\mu_{n}\left(T_{n}\right)=\frac{\mu_{0}}{{\left[\sqrt{1+\gamma^{2}{\left(T_{n}-T\right)}^{\beta }}+\gamma {\left(T_{n}-T\right)}^{\beta /2}\right]}^{\beta /2}}$$

where *γ* is given by *γ* = (1/2)(3*µ*_0_/2*eτ*_*ε*_*v*_s_^2^)^*β*/2^.

For the TE model, the same procedure is applied, but the equation for *μ*_*n*_(*T*_*n*_) can only be solved numerically. It is also necessary to note that the above approach for derivation of *μ*_*n*_(*T*_*n*_) is internally contradictive since it relies on the energy balance equation in a homogeneous steady-state electric field. Unfortunately, there is not much that can be done to overcome this problem. A rigorous approach to obtain *μ*_*n*_(*T*_*n*_) in the case of strong inhomogeneous instantaneous fields would require a full solution of the microscopic Boltzmann equation, which is practically an impossible task for realistic devices. The other option is to use the Monte Carlo method, but this is again impractical for many real devices. There were also some suggestions [26,27] on how to include the non-local effects in calculating non-equilibrium kinetic coefficients, like mobility or diffusivity, but these approaches have not been implemented yet into numerical models.

**Figure 3:**
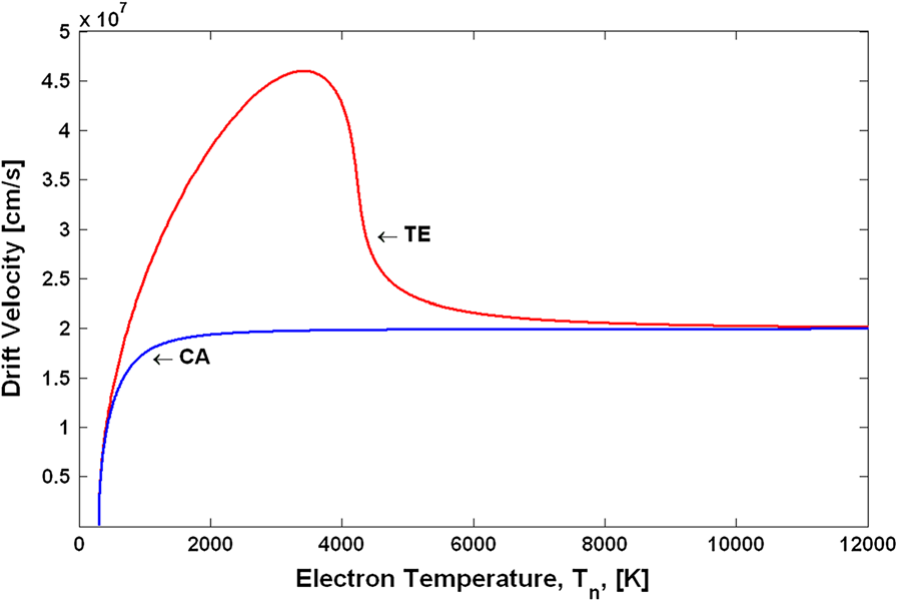
Drift velocity of GaN as a function of the electron temperature.

**Figure 4:**
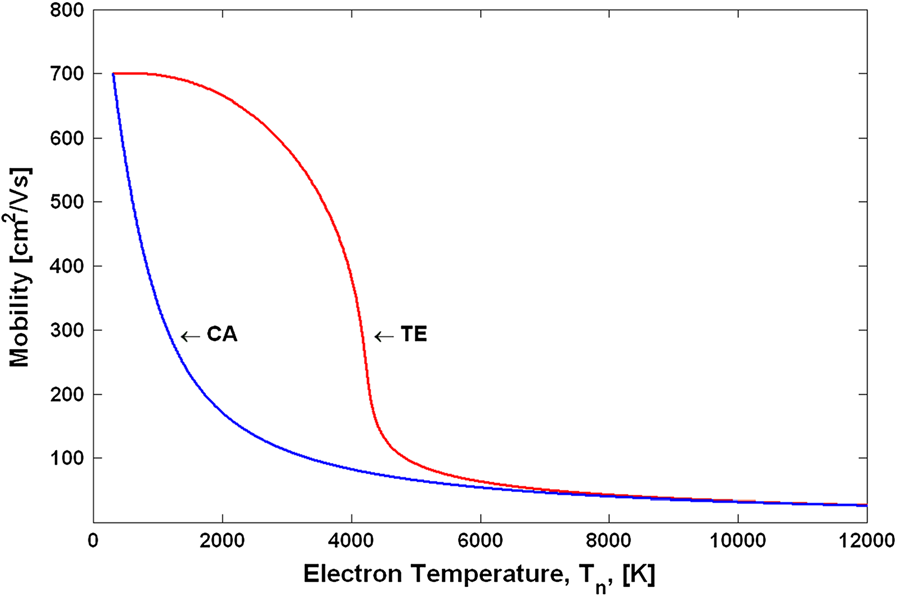
Electron mobility of GaN as a function of the electron temperature.

**Figure 5:**
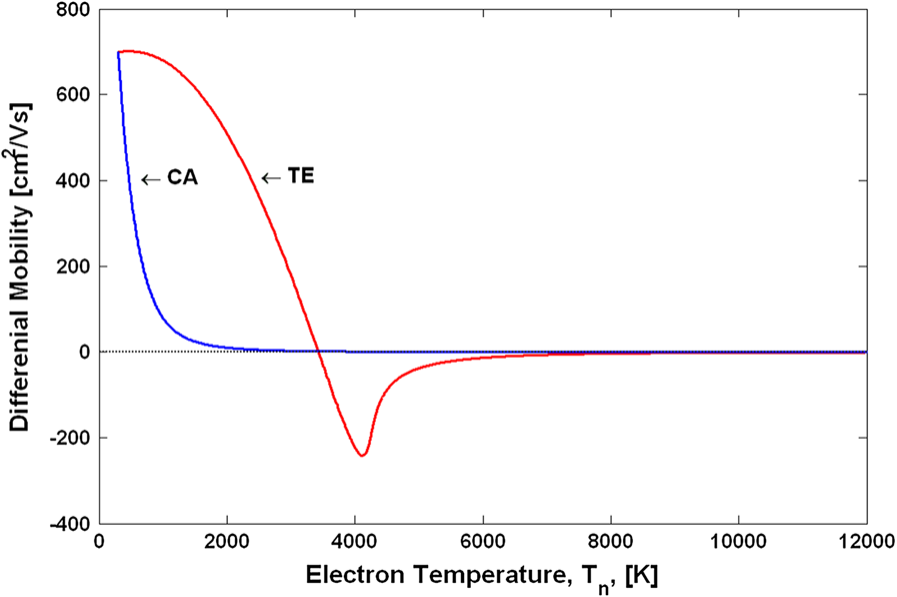
Differential mobility of GaN as a function of the electron temperature.

Using the above procedure, we have calculated the electron temperature-dependent mobilities and the v-F characteristics for the TE and CA models. Figure 3 shows the v-F characteristics (drift velocity as a function of the electron temperature), and Figure 4 shows the *μ*_*n*_(*T*_*n*_) dependences. Note that due to the peculiarities of the approach in deriving the *μ*_*n*_(*T*_*n*_) dependences, as explained above, the shape of the differential mobilities as functions of the electric field is the same as those shown in Figure 2 for the TE and CA models. The differential mobilities as functions of the electron temperature are shown in Figure 5. Using the obtained *μ*_*n*_(*T*_*n*_) dependences in the basic equations (Equations 1, 2, 6, 7 and 8) for the HD model, one can simulate the spatial-temporal distributions of the carrier density *n*(*xt*), the electric field *E*(*xt*) and the electron temperature *T*_*n*_(*xt*). This is the end result of the HD model. Note that in a spatially inhomogeneous system, the local electric field *E*(*xt*) and the local electron temperature *T*_*n*_(*xt*) are not related anymore to each other by a single-electron local balance equation *eµ*_*n*_(*T*_*n*_)*E*^2^ = (3/2)*k*_0_*T*_*n*_ − *T*/*τ*_*ε*_, as can be seen from the more general energy balance equation (Equation 6). Thus, it may be said that it is the energy balance equation which is in general responsible for a non-local dependence of the electron mobility on the electric field. The relationship between the differential mobility and the electric field has in general non-local character as well. Taking into account particular importance of the differential mobility in devices with the transferred electrons [1,9], like Gunn diodes, it is clear that the aforementioned non-locality must have serious influence on the operation modes, as will be confirmed later by the simulation results.

### Transient effects from the DD and HD models

A short bias pulse *U*(*t*) of amplitude *U*_0_ = 28 V (corresponding to the electric field strength of 140 kV/cm, which is above the threshold field) with a very steep rise time was applied to a 2-μm GaN sample using a photoconductive switch in the circuit which was described earlier. We have chosen a trapezoidal-shaped pulse with plateau duration equal to 1 ps. The pulse rise and fall times were varied between 0.1 and 10 ps in different simulations.

**Figure 6:**
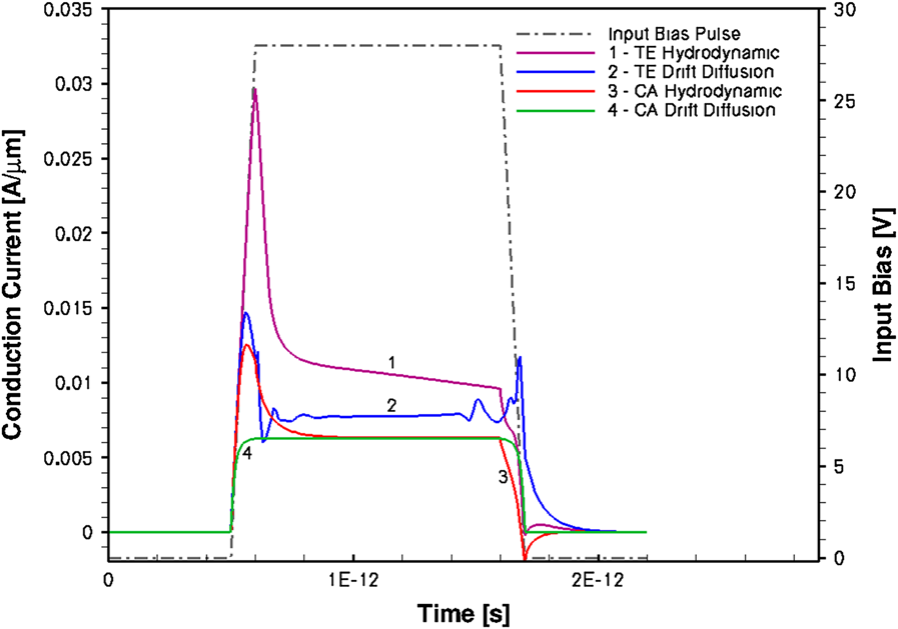
Transient conduction currents of GaN sample from DD and HD modelling.

The results of the simulations for the conduction current *I*_*n*_(*t*) response are shown in Figure 6 for the HD model (curves 1 and 3) and the DD model (curves 2 and 4) using the TE and CA mobility models. As can be seen from Figure 6, the current from the DD model (curve 2) simply follows the v-F characteristics shown in Figure 1: it peaks at exactly the threshold field when the drift velocity is at its maximum, then it decreases following the v-F curve branch with the NDM, and finally saturates at the bias *U*_0_ = 28 V. Regarding the DD results, we have to point out that when the TE mobility model was used, there was a severe convergence problem at the point where the bias *U*(*t*) reaches the plateau. This problem persisted even when the rise time was increased to 10 ps. As a result, the calculated output current *I*_*n*_(*t*) had a correct shape but somewhat lower than the expected value at the plateau. Further investigation of this problem has shown that it is most likely due to discontinuity of the derivative *dU*(*t*)/*dt* at the above point. (For the DD with the CA mobility model (curve 4 in Figure 6), there was no convergence problem, and *I*_*n*_(*t*) exactly follows the v-F curve.) When we used a Gaussian pulse as *U*(*t*), the same DD simulation with the TE mobility model runs smoothly. Thus, we may conclude that the DD-simulated transient current responses *I*_*n*_(*t*) in GaN homogeneously doped bulk samples exactly follow the v-F curves for both TE and CA mobility models, with no sign of any Gunn-type instabilities developing in the system in the transient regime.

The latter result can be explained as follows: The propagating Gunn domains are generated in the sample if the doping-length product *n*_0_*L* is larger than some threshold value, *n*_0_*L* > (*n*_0_*L*)_0_[1,9]. For GaAs, the parameter (*n*_0_*L*)_0_ ≈ 10^12^ cm^−2^[1,9]. For GaN, this parameter is considerably larger because the NDM in GaN is about an order of magnitude smaller, as was shown earlier. For the zinc-blende GaN with the parameters considered in this paper, we obtained (*n*_0_*L*)_0_ ≈ 3.7 × 10^12^ cm^−2^, which is somewhat larger but close to the value of 2.5 × 10^12^ cm^−2^ obtained in [15]. In our sample, the doping-length product is *n*_0_*L* = 2 × 10^13^ cm^−2^. Therefore, the criterion for the Gunn domain instability is well satisfied. (Note that if the opposite criterion is satisfied, i.e. *n*_0_*L* < (*n*_0_*L*)_0_, then the accumulation layer instability may develop [1,9]. In this case, the layer with the excess of electrons (negative space charge) and no depletion layer (positive space charge, as is the case for Gunn domains) can be generated at the cathode and propagate to the anode.) The above criterion is, however, the necessary but not the sufficient condition for the domain instability since it was obtained on the assumption of the existence of a stable propagating domain. The domain still needs to be generated at the cathode. This in turn requires some built-in perturbation (e.g. the doping notch) in the sample or some other physical processes which can stimulate the domain nucleation. Since our sample is ideally homogeneous, the required conditions for the domain nucleation are not satisfied in the DD model. The short available time, approximately 1 ps for our bias pulse, is also the reason for preventing the instability development. However, this is not the main reason for the absence of any sign of the instability. We have analysed the spatial-temporal distributions of the carrier densities and the electric fields in the sample obtained from the DD model and found no sign of any deviation from the homogeneous state, even when the pulse duration has been considerably increased. We want to stress that the above observation is not necessarily what may take place in a real device. We merely want to point out that in the samples under consideration (about 2- to 3-μm-long GaN samples doped at 10^17^ cm^−3^), the transient response in the DD model with the TE mobility does not show any sign of instability in spite of the fact that the necessary criterion for Gunn domains is satisfied. This point will be further discussed when we will analyse the DC-driven GaN devices.

The situation is completely different with the HD model. As can be seen from Figure 6 (curve 1), the transient conduction current response is considerably larger than in the DD model (curve 2). This was also checked for the Gaussian bias pulses *U*(*t*) of different duration. It was found that the HD model consistently gives larger transient currents. The same is true when the CA mobility model is used (curves 3 and 4), although the peak current for the TE model is considerably larger than that for the CA model.

**Figure 7:**
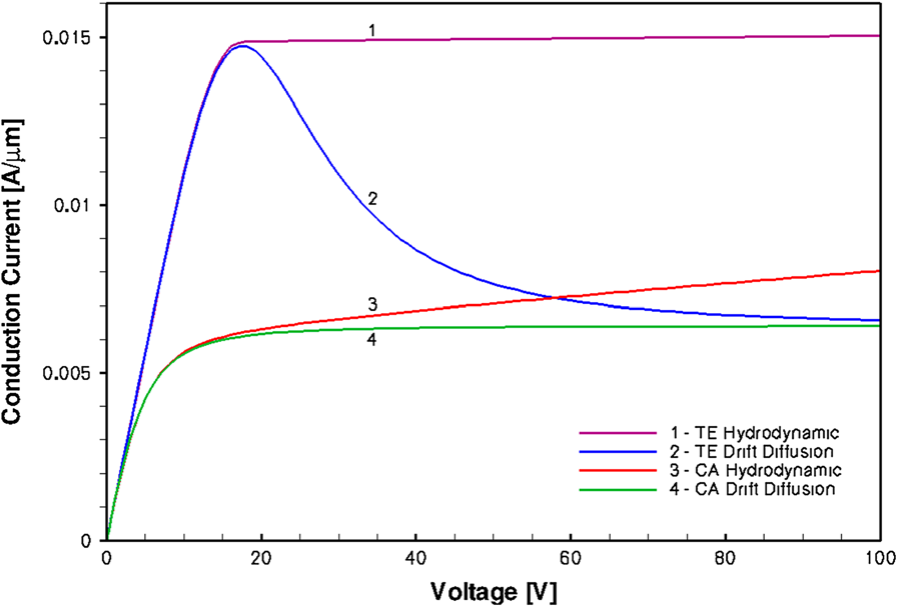
**DC steady-state *I*-*V *characteristics of GaN sample.**

The hint to the physical reason behind these differences can be seen from the steady-state simulations of the same device, the results of which are shown in Figure 7. The DC *I-V* characteristics are remarkably different in the DD and HD models.

There are at least two physical reasons why the DC and transient currents in the HD model should be larger than those in the DD model. Both of these reasons are most likely to be present in the real Gunn diode devices, and thus, the HD model should give a correct description of the underlying physical processes in real devices which are not included in the DD model.

**Figure 8:**
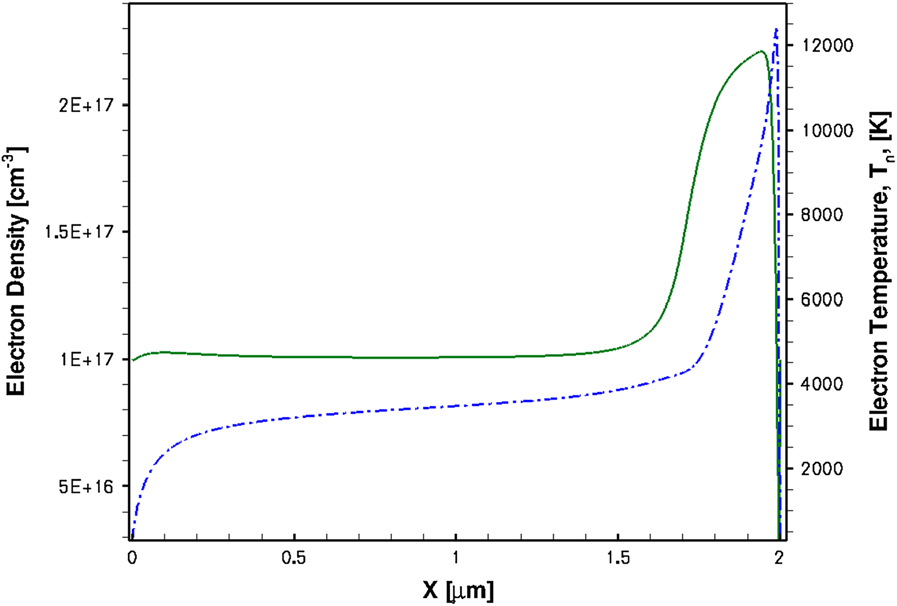
Spatial distributions of electron density (solid line) and electron temperature (dash-dotted line) in steady state.

First of all, the saturation of the DC current in the HD model (curve 1 in Figure 7) clearly shows that the *IV* characteristics do not follow the v-F dependence, and the negative differential resistance *R*_d_^−1^ = *dI*/*dU* is not observed here in spite of the presence of the NDM (Figure 5). The reason why the DC current is saturated is because of the excess carrier injection from the cathode, as can be seen from Figure 8. The increase of the carrier density in the sample when the electric field increases exactly compensates the drift velocity decrease in the TE model. (Note that the HD model with the CA mobility also shows some carrier excess injection, as it is evidenced by the absence of the current saturation in the *IV* characteristics, curve 3 in Figure 7.) What is seen in Figure 8 is the stable anode-trapped domain which was first predicted in [28] and theoretically investigated in [29] (see also Chapter 4 in [9] and the references therein). As it was shown in [29] and [8] at some conditions, the anodic domain can be formed (trapped) either from the propagating Gunn domains or from the propagating accumulation layers. (We will show later that it is the latter variant which takes place in our devices and will explain the physical reason.) The injected excess space charge results in the inhomogeneous electric field distribution, as confirmed by inhomogeneous electron temperature profiles in Figure 8.

**Figure 9:**
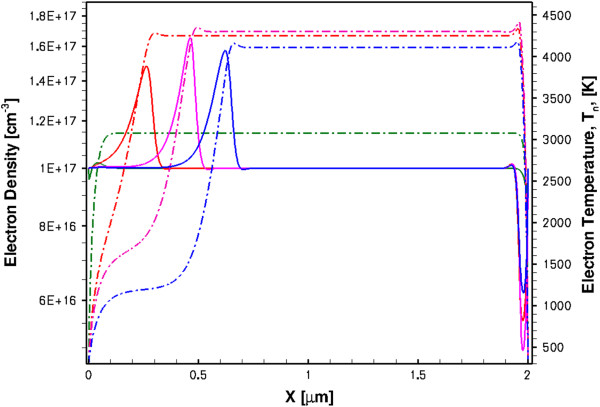
Spatial distributions of electron density and electron temperature at different times in transient regime.

The space charge injection in the HD model seen in the DC regime is also present in a transient regime, as it is shown in Figure 9 (solid lines). In the transient regime, the accumulation layer is not trapped; it is nucleated at the cathode at the very beginning of the bias pulse, and it continues growing due to the excess carrier injection from the cathode when it propagates to the anode. As a result of the carrier density increase in the active region, the current also increases, as is seen in Figure 6 (curve 1). Since the sample must remain globally neutral, an equal amount of electrons are extracted at the anode leading to the depletion region near the anode, as can be seen from Figure 9. The transient electron temperature distributions are also shown in Figure 9 (dash-dotted lines). The propagating accumulation layer splits the active region into two parts - the high-field front region between the layer and the anode, and the rear low-field region between the cathode and the layer. The electrons are considerably hotter in the front region. Since the accumulation layer is moving, the split is dynamic and the electron temperature profiles are changing during the accumulation layer transit time.

The second effect which contributes to the increase of the transient current response is the velocity overshoot effect in the regions very close to the contacts. During a very short time when the bias pulse is applied, the carrier velocities in these regions are higher than the peak velocity. This effect clearly appears in the HD models, but is completely absent from the DD models. The overshoot is absent in the DC regime. For the DD model, one can also see the difference between the DC current at *U* = 28 V (curve 2 in Figure 7) and the transient current corresponding to the plateau when the bias is also *U*_0_ = 28 V (curve 2 in Figure 6). There is no physical reason for this difference. As was explained earlier, it is due to the convergence problems in the DD transient simulations for a trapezoidal bias pulse which in turn deteriorated the numerical accuracy of the simulation.

The aforementioned two effects, the space charge injection and extraction at the electrodes and the velocity overshoot near the contacts, result in a very short (approximately 0.2 ps) high-peak conduction current pulse (curve 1 in Figure 6). The spectral bandwidth of this transient current is approximately 5 THz. If the sample is monolithically integrated with a suitable antenna as in [11], the setup can generate MW and THz waves. We also observed the pulses of the displacement currents (not shown in Figure 6) which should also generate radiation in the same spectral region.

The generated current pulses obviously depend on the rise and fall times and the shape of the input bias *U*(*t*), as was confirmed by the simulations. We have also investigated transient response to a Gaussian input *U*(*t*) with amplitude *U*_0_ = 28 V and full width at half maximum (FWHM) of 0.5 and 1 ps. The results were very different for the DD and HD models with considerably larger currents in the HD model. Our DD results were very similar (except the absence of the overshoot) to the results obtained in [5] where the external MW Gaussian pulses were incident on the sample and the current response was calculated using the HD model. The results are similar because in both cases the electric field distributions in the samples were homogeneous and the current responses simply follow the v-F characteristics, although the reasons for the homogeneous field distributions are completely different. In [5], this was due to the design of the experimental setup; in our case, the reasons are in the physical limitations of the DD model (in particular, its local character).

### Large-signal analysis of MW and THz operation of GaN-based Gunn diodes

A non-trivial behaviour of transient current responses revealed in the HD modelling with two prominent features (space charge injection and the velocity overshoot, as was discussed in the previous section) and complete absence of these effects in the DD modelling clearly indicate that the results from the DD and HD models of a Gunn diode oscillator may be profoundly different. In some cases, the DD model may give a qualitatively false description of the modelled device. This may happen, for example, in cases where the above two effects play a crucial role. The supercritically doped GaN sample operating in the regimes with NDM is, in our opinion, one such case. In this section, we investigate operation of a voltage-controlled GaN-based oscillator using the same sample as before.

When the voltage step of 28 V is applied to the sample, the corresponding electric field of 140 kV/cm is above the threshold; thus, the diode may in principle operate in one of the oscillatory modes. As was shown earlier, there exist non-oscillatory steady states for 28-V DC bias, although these states are very different in the DD and HD models. The existence of the steady state does not mean yet that the system will end up in this state when it evolves in time in response to the bias step. The steady state may be unstable with respect to small perturbations.

In spite of the existence of a spatially homogeneous steady state in the DD model, our simulation of the step bias response had severe convergence problems and did not produce any state with regular oscillations. We have varied the rise time of the input pulse from 1 ps to 1 ns, but the only output from the DD model was the chaotic current oscillations with very poor convergence. These oscillations start either at the point where the maximum bias is reached (if the bias rise time was chosen very short) or at the point corresponding to the threshold bias (for long rise time). Apparently, the fact that the system is ideally homogeneous with no perturbations (e.g. notches) plays a crucial role for the DD model. As a result, neither the Gunn domains nor the accumulation layers can nucleate at the cathode, and they did not appear in the simulation.

**Figure 10:**
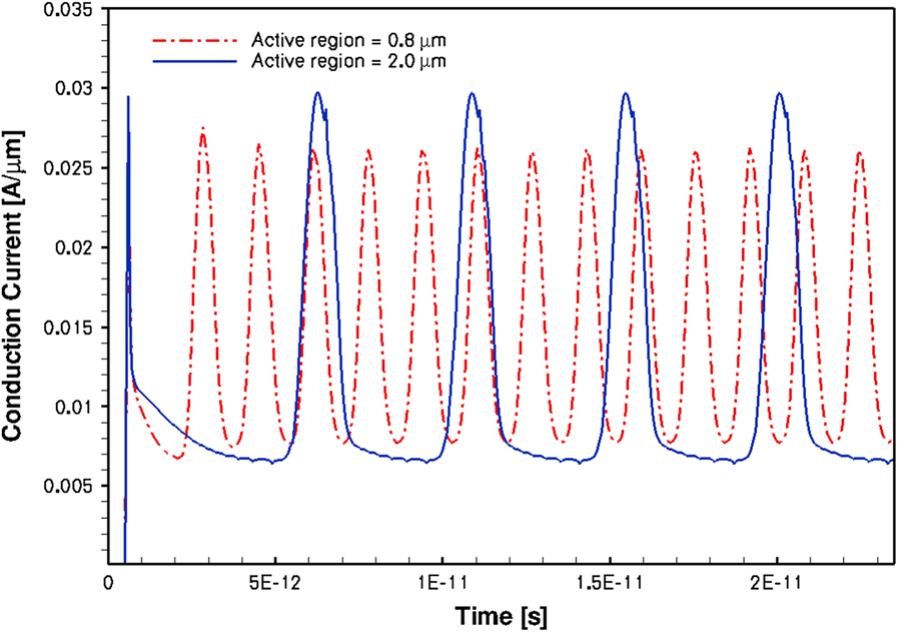
Oscillations of conduction current in 2- and 0.8-μm-long GaN at 28- and 11.2-V biases, respectively.

**Figure 11:**
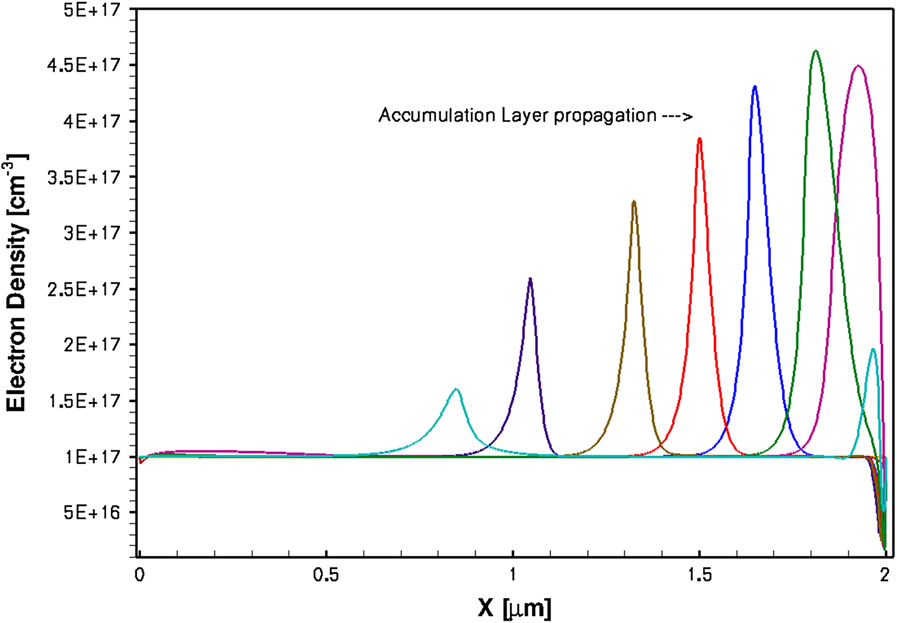
Accumulation layer propagation in the 2-μm sample at 28-V bias.

**Figure 12:**
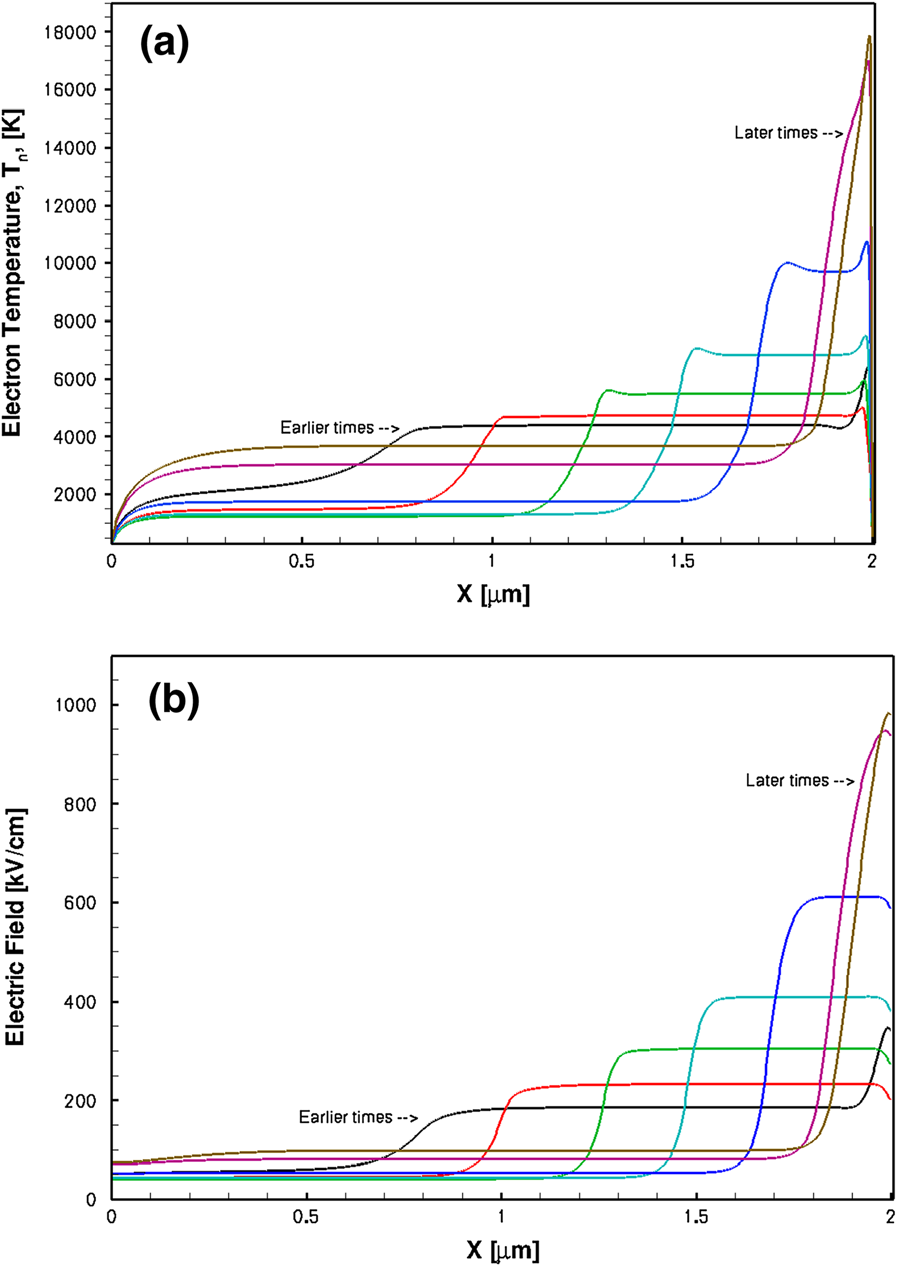
**Electron temperature profiles (a) and electric field distributions (b).** Both follow the accumulation layer propagation from the cathode to the anode.

A completely different behaviour was found in the HD modelling. The diode was oscillating in the accumulation layer mode producing very sharp conduction current pulses (FWHM approximately 1 ps) at a repetition frequency of about 0.25 to 0.3 THz. These results are shown in Figure 10. The displacement current has similar parameters, and it is in anti-phase with the conduction current. The current oscillation frequency is determined by the sample length *L*, so it can be easily increased using shorter samples, as shown by the dash-dotted line in Figure 10 where *L* = 0.8 μm. There are also other design possibilities to increase the oscillation frequency. The corresponding oscillation frequency for the latter sample was approximately 0.63 THz, and the FWHM for the conduction current spikes is approximately 0.5 ps. The fact that the propagating perturbations are not Gunn domains is supported by the results in Figures 11 and 12a,b for the spatial-temporal distributions of electron density, electron temperature and electric field, respectively. As it is seen from the figures, there is no depletion region and the propagating perturbations are pure accumulation layers. It is also instructive to observe from Figure 12a that the electron gas in the studied GaN sample is in a highly non-equilibrium state with the maximum electron temperature near the anode of about 18,000 K. This carrier temperature corresponds to the mean electron energy of about 1.6 eV. The inter-valley separation in zinc-blende GaN has approximately the same value, so the hot electrons are capable of taking part in the inter-valley transitions. This in turn results in the NDM transport regime as described by the phenomenological Equation 4 for the electron mobility. Therefore, the very high electron temperature is necessary for the electrons to exhibit the NDM in GaN samples. For comparison, in GaAs, the corresponding carrier temperature is substantially lower than in GaN due to considerably lower inter-valley separation (0.3 eV in GaAs).

There are two striking features of the above results. The first feature is related to the physical origin of the accumulation layer nucleation. The layers are originated at the cathode due to the non-local effects. When the electrons are injected from the cathode (or extracted from the sample at the anode), they perturb the electric field distribution. Since in the HD model the relation between the mobilities and the field is non-local, the mobility does not follow the field. In addition, the drift velocity near the electrodes is not related to the mobility by the usual relationship *v*_d_ = *μ*_*n*_*E* because of the velocity overshoot effect. (Near the cathode, the overshoot probably takes place outside of the so-called ‘dead-zone’ [18], where the electric field is still low because of the boundary condition *E*(*x* = 0,*t*) = 0.). Since these effects are completely absent in the DD model due to its local nature, this explains why the DD model is not able to tackle the accumulation layer regime in our case.

We want to make it clear that this does not mean that the DD model cannot be used in principle in the accumulation layer regime (see, e.g. [30,31]). The operation modes of the Gunn diodes depend on the other parameters of the device, and this dependence is in general of a very complex nature. Depending on a particular combination of the parameters, the DD model may describe fairly well the accumulation layers in other devices. For example, the short length of our device is one of the detrimental factors for the DD model. Even more, when we decreased the device length below approximately 0.8 μm, we found in the HD model that the accumulation layers are trapped at the anode, giving a stable anodic domain [28], and the oscillation regime was not observed. The physical reason for this was that the accumulation layer width becomes comparable with the sample length and the layer fills out the whole sample and cannot propagate.

Another interesting result is the propagation speed of the accumulation layers. As it can be calculated directly from Figure 11, the propagation speed of the layers is about 5 × 10^7^ cm/s, which is larger than the peak drift velocity of the electrons. This result has been known since the discovery of the accumulation layer regime and was first discussed in [31] and theoretically obtained in [30]. The result is of great practical importance as it shows that the accumulation layer mode of the Gunn diode oscillator may provide higher oscillation frequencies than the Gunn domain mode since the latter usually propagates at a speed lower than the peak drift velocity [9] (most likely, the Gunn domain speed is close to the electron saturation velocity).

We have compared our results with the HD simulations of GaN-based Gunn oscillators in [15]. Our devices have some design differences, namely the length in [15] is 3 μm, and there are also *n*^*+*^ contact regions; the rest of the parameters are very close. The oscillator in [15] was placed in the external resonant cavity. The authors of [15] believe that the operation mode of their diode was the Gunn transit domain regime since the necessary criterion *n*_0_*L* > (*n*_0_*L*)_0_ was satisfied. However, as we explained earlier, the Gunn domain criterion is the necessary but not the sufficient condition of the operation mode. It is difficult to directly compare our results, given that in [15] no spatial distributions of electric fields or carrier densities in the device were presented to validate the assumption. We think that the actual operation regime in [15] is the accumulation layer mode, for the reasons outlined above, and our additional simulation for exactly the same device design and parameters as in [15] (but without the external cavity) did support this conclusion.

## Conclusions

In summary, we have carried out a detailed study of high-field transient and DC transport in GaN-based Gunn diode oscillators using the commercial simulator Sentaurus Device. In the first part of the work, we have investigated in depth the applicability of the DD and HD models to high-speed, high-frequency devices. The results of the simulation from these two models are compared and evaluated. It is shown, for a highly homogeneous device based on a short (2 μm) supercritically doped (10^17^ cm^−3^) GaN specimen, that the DD model is not able to correctly take into account some essential physical effects which determine the operation mode of the device. The use of the DD model in such cases will most likely produce incorrect results. We demonstrate that the HD model is ideally suited to solve such problems due to its ability to incorporate non-local effects.

We show that the velocity overshoot near the device's contacts and space charge injection and extraction play a crucial role in defining the operation mode of highly homogeneous short diodes in both the transient regime and the voltage-controlled oscillation regime. The transient regimes are studied for an in-series connected Gunn diode and a photoconductive switch when a picosecond bias pulse is directly applied to the contacts. The conduction current responses are fundamentally different in the DD and HD models. The DD current simply reflects the v-F characteristics, and the sample remains in a completely homogeneous state. In the HD model, the transient current pulse with FWHM of approximately 0.2 ps is about twofold larger than that in the DD model due to the carrier injection (extraction) into (from) the active region and the velocity overshoot. The electron gas is characterized by highly inhomogeneous distributions of the carrier density, the electric field and the electron temperature. It is suggested that the generated ultra-short current pulses may be used to feed the integrated antenna radiating in a wide range including the THz and sub-THz range. The simulation of the DC steady states of the diodes using the DD and HD models shows very different results. The HD model shows the trapped anodic domain, while the DD model completely retains all features of the v-F characteristics in a spatially homogeneous gas.

Finally, the simulation of the voltage-controlled oscillator shows that it operates in the accumulation layer mode generating MW signals at 0.3 to 0.7 THz. The FWHM of the oscillating conduction current pulses was between 0.5 and 1 ps. In spite of the fact that the known criterion of a Gunn domain mode *n*_0_*L* > (*n*_0_*L*)_0_ was satisfied, no Gunn domains were observed. This phenomenon is explained by the fact that the above condition is the necessary but not the sufficient condition. A comparison of our results with other published works on GaN-based Gunn diodes shows that some of the previously reported Gunn domain regimes may in fact be the accumulation layer modes of the device operation.

## Competing interests

The authors declare that they have no competing interests.

## Authors’ contributions

EM participated in the design of the study and carried out the device simulations. NZ conceived of the study, analysed critically the simulation results and drafted the manuscript. NB revised the manuscript and gave final approval of the version to be published. All authors read and approved the final manuscript.
